# Long term N-acetylcysteine administration rescues liver steatosis via endoplasmic reticulum stress with unfolded protein response in mice

**DOI:** 10.1186/s12944-020-01274-y

**Published:** 2020-05-25

**Authors:** Ching-Chou Tsai, Yu-Jen Chen, Hong-Ren Yu, Li-Tung Huang, You-Lin Tain, I-Chun Lin, Jiunn-Ming Sheen, Pei-Wen Wang, Mao-Meng Tiao

**Affiliations:** 1 Department of Obstetrics and Gynecology, Kaohsiung Chang Gung Memorial Hospital and Chang Gung University College of Medicine, Kaohsiung City, Taiwan; 2 Graduate Institute of Clinical Medicine, Kaohsiung Medical University, Kaohsiung City, Taiwan; 3 Department of Obstetrics and Gynecology, Chiayi Chang Gung Memorial Hospital, Chiayi County, Taiwan; 4 Department of Pediatrics, Kaohsiung Chang Gung Memorial Hospital and Chang Gung University College of Medicine, Kaohsiung City, Taiwan; 5 Department of Pediatrics, Chiayi Chang Gung Memorial Hospital, Chiayi County, Taiwan; 6 Department of Internal Medicine, Kaohsiung Chang Gung Memorial Hospital and Chang Gung University College of Medicine, Kaohsiung City, Taiwan

**Keywords:** Acetylcysteine, Steatosis, Liver, ER stress

## Abstract

**Background:**

Fat accumulation in the liver contributes to the development of non-alcoholic fatty liver disease (NAFLD). N-acetylcysteine (NAC) is an antioxidant, acting both directly and indirectly via upregulation of cellular antioxidants. We examined the mechanisms of liver steatosis after 12 months high fat (HF) diet and tested the ability of NAC to rescue liver steatosis.

**Methods:**

Seven-week-old C57BL/6 (B6) male mice were administered HF diet for 12 months (HF group). Two other groups received HF diet for 12 months accompanied by NAC for 12 months (HFD + NAC(1–12)) or 6 months (HFD + NAC(1–6)). The control group was fed regular diet for 12 months (CD group).

**Results:**

Liver steatosis was more pronounced in the HF group than in the CD group after 12 month feeding. NAC intake for 6 or 12 months decreased liver steatosis in comparison with HF diet (*p* < 0.05). Furthermore, NAC treatment also reduced cellular apoptosis and caspase-3 expression. In the unfolded protein response (UPR) pathway, the expression of ECHS1, HSP60, and HSP70 was decreased in the HFD group (*p* < 0.05) and rescued by NAC therapy. With regards to the endoplasmic reticulum (ER) stress, Phospho-PERK (p-PERK) and ATF4 expression was decreased in the HF group, and only the HFD + NAC(1–12), but not HFD + NAC(1–6) group, showed significant improvement.

**Conclusion:**

HF diet for 12 months induces significant liver steatosis via altered ER stress and UPR pathway activity, as well as liver apoptosis. NAC treatment rescues the liver steatosis and apoptosis induced by HF diet.

## Background

According to the World Health Organization, there are an estimated 500 million and 1.5 billion obese and overweight/obese people worldwide, respectively [1]. Obesity is associated with chronic inflammation, and numerous proinflammatory cytokines promote the development of non-alcoholic fatty liver disease (NAFLD) [2]. NAFLD is defined as the presence of steatosis in more than 5% of the hepatocytes, determined by histological analysis. Furthermore, the disorder is associated with excessive hepatic fat accumulation and insulin resistance [3, 4].

The “multiple hit pathogenesis” hypothesis, proposed to explain the origin of NAFLD, is multifactorial, including genetic, epigenetic, metabolic, and environmental parameters. These factors lead to the accumulation of fat, like triglycerides, in hepatocytes, rendering them more susceptible to certain stress types, such as oxidative stress, ATP depletion, and endotoxins. These finally cause inflammation, cellular death, and fibrosis [5]. Lipotoxicity results from the excess accumulation of fat in the liver, leading to mitochondrial dysfunction and endoplasmic reticulum (ER) stress [6]. Mitochondrial dysfunction results in reactive oxygen species (ROS) overproduction, causing abnormal respiration, and then stimulates NAFLD development [7].

N-acetylcysteine (NAC), a precursor of de novo glutathione (GSH) biosynthesis, acts as an antioxidant both directly and indirectly by increasing cellular antioxidant levels, especially in hepatic tissue. Since liver is susceptible to oxidative, free radical injury and inflammation, leading to NAFLD, NAC may play a role in preventing liver damage [8–11]. In addition, NAC was also used for early psychosis, enhancing performance of elite sports, decreasing alveolar inflammation and restoring GSH synthesis [11]. NAC combined metformin rescue liver steatosis is in the human study [12]. The sole use of NAC in the human liver steatosis is still unknown. Though, Yang’s study mentioned about the NAC effect in the rescue hepatocyte cells (HepG2) fat [13]. The long term effect of NAC in the liver steatosis is still unknown.

According to previous studies, postnatal high fat (HF) diet can lead to NAFLD through mechanisms including oxidative stress, inflammation, and nutrient-sensing signals [14–16]. The mechanism via unfolded protein response (UPR) pathway and ER stress in liver steatosis is still unknown. Here, we aimed to elucidate the effects of NAC on liver steatosis induced by 12 months HF diet.

## Materials and methods

### Animals and tissue preparation

Seven-week-old C57BL/6 (B6) male mice were housed in the animal care facility of the Chang Gung Memorial Hospital, Kaohsiung, Taiwan under a 12 h light/dark cycle; the lights were turned on at 7 a.m. The experimental animals were allowed ad libitum access to water and food and underwent a 12 months treatment. Mice were divided into five groups: (1) chow diet group [(CD), 3.85 kcal/g dry wt, 19.2 g/100 g protein, 67.3 g/100 g carbohydrate, and 4.3 g/100 g saturated fat]; (2) chow diet with 10 mMNAC (Sigma-Aldrich/A9165**,** Louis, MO, USA) dissolved in water administered for 12 months (CD + NAC); (3) high-fat diet group[(HF), 5.56 kcal/g dry wt, 23 g/100 g protein, 35.5 g/100 g carbohydrate, and 35.8 g/100 g saturated fat mostly in the form of lard (58 kcal% fat, Research Diets/D12331, New Brunswick, NJ, USA)] after weaning; (4) HF with 10 mM NAC after weaning for 12 months (HF + NAC(1–12)); (5) HF supplemented with 10 mM NAC for the first 6 months of treatment after weaning (HF + NAC(1–6)). Mice were anesthetized with a muscle injection zoletil and rompun (1:2 mixture); the liver was immediately dissected out, sectioned on an ice-plate, and stored for future analysis.

### Hepatic triglyceride assay

Liver tissues (350–400 mg) were homogenized and centrifuged at 10,000×g for 10 min at 4 °C. The supernatant was assayed using a triglyceride by triglyceride colorimetric assay kit (Cayman, 1,001,303, Ann Arbor, Michigan, USA), according to the manufacturer’s instructions.

### Hematoxylin-eosin (H&E) staining

The livers were dissected and fixed in 4% paraformaldehyde at 4 °C overnight. Next, the fixed tissues were dehydrated in gradient ethanol (70, 75, 85, 90, 95, and 100%), hyalinized in xylene, and embedded in paraffin wax at 55 °C. Sections were cut at 3 μm and stained with H&E Stain Kit (ScyTek Laboratories, West Logan, USA) following the manufacturer’s instructions. A microscope (Leica DMI-3000), equipped with a digital camera, was used to observe the histological lesions. The H&E images were quantified lipid accumulation in liver by image J [17, 18]. The procedure briefly described as the image first was convered to an 8-bit gray-scale image then black-white inverted. The black-white inverted image was adjusted threshold of the gray scale to remove inter-hepatocyte structures not indicating lipid droplet features, followed by particle analysis.

### TdT-mediated dUTP-biotin nick end labeling (TUNEL) assay

Tissues were immersed in 4% paraformaldehyde in PBS and fixed overnight at 4 °C. Fixed tissues were paraffin-embedded, cut into 3 μm thick transverse sections, and mounted on slides. An apoptosis detection kit (Roche, 11,684,817,910, Mannheim, Germany) was used according to the manufacturer’s instructions. Sections were visualized with 3, 3-diaminobenzidine tetrahydrochloride and counterstained with Gill’s hematoxylin. Cells were counted from randomly selected high-power fields (200×) from each section under light microscopy, and the rates of TUNEL-positive cells were calculated. A total of 500 hepatocytes from each mice were used to count positively stained cells.

### Western blot

Livers were dissected and subsequently frozen in liquid nitrogen. The tissue of each liver was homogenized in lysis buffer (cat. no. 17081; iNtRON Biotechnology, Seongnam, Korea) and centrifuged at 14,000×*g*. Protein (65 μg) from the supernatant of each sample was separated by SDS-PAGE and transferred onto polyvinylidene difluoride (PVDF) membranes. Membranes were blocked in TBST buffer containing 10% non-fat milk for 1 h at room temperature. Immunoblotting was performed by incubating the blocked membrane overnight at 4 °C with the following antibodies: monoclonal anti-cleaved caspase-3 (Cell signaling/#9661, Danver, MA, USA), anti-phospho-protein kinase RNA-like endoplasmic reticulum kinase (p-PERK) (Cell signaling/#3179), activating transcription factor 4 (anti-ATF4)(Cell signaling/#11,815), anti-HSP70 (Cell signaling/#4872), anti-HSP60 (Cell signaling/#4870), anti-ATP-dependent Clp protease proteolytic subunit (ClpP)(Abcam/ab124822, Cambridge,MA, USA), anti-SREBP1 (Thermo/PA1–337, Waltham,MA, USA), anti-enoyl-CoA hydratase (ECHS1) (Protein- tech/11305,Rosemont, IL, USA),and anti-GAPDH antibody (Thermo/MA-15738). The membranes were then incubated with secondary HRP-conjugated anti-rabbit (1:5000; Jackson Immuno Research, West Grove, PA USA) or anti-mouse antibody (1:10,000; Jackson Immuno Research) for 1 h at room temperature. Western blots were visualized using an ECL kit (Perlcin Elmer In. /NEL 105001EA, Boston, MA, USA). The quantification of western blot was performed with Quantity one software version 4.52 (Bio-Rad) to select and determine the background-subtracted density of the bands in all blots. The results were normalized to that of GAPDH expression [19].

## Statistical analysis

SPSS 15.0 for Windows was utilized for statistical analysis. For most parameters, analysis of variance (ANOVA) with a Bonferroni post hoc test was used. The data are presented as mean ± standard error of the mean (SEM). The level of statistical significance was set at *P* < 0.05.

## Results

### Body, liver weight and triglyceride

**Fig. 1:**
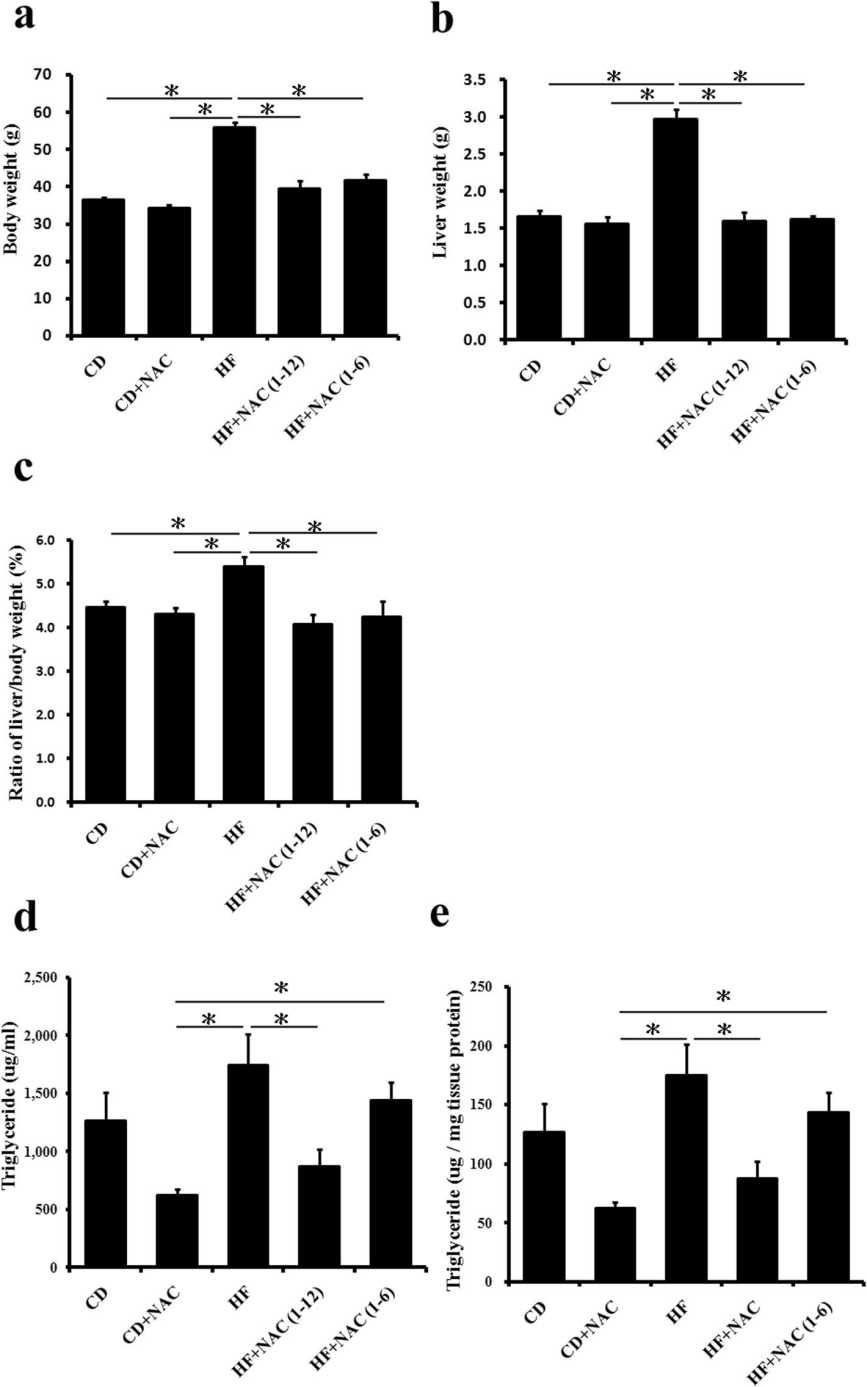
Body weight, liver weight and triglyceride accumulation in liver were significantly increased in the high fat diet group (HF), and it could be retarded with NAC treatment. **a** Determination of body weight, **b** liver weight and **c** percentage ratio of liver/body weight. **d** Determination of triglyceride in liver tissue extraction, **e** then conversed to triglyceride content (μg) per milligram of liver tissue. All values are expressed as mean ± SEM (*n* = 6). *P < 0.05. The letters represented different groups (CD for chow diet group; CD + NAC for chow diet and NAC intervention 1–12 months group; HF for high fat diet group; HF + NAC (1–6) for high fat diet and NAC intervention 1–6 months group; HF + NAC (1–12) for high fat diet and NAC intervention 1–12 months group)

The body weight increased in the HF group, and NAC treatment significantly reversed this effect (Fig. 1a). Furthermore, NAC decreased the liver weight gain induced by HF diet (Fig. 1b). The triglyceride content increased in the HF group and NAC treatment decreased the content (Fig. 1c, d).

### Liver steatosis

**Fig. 2:**
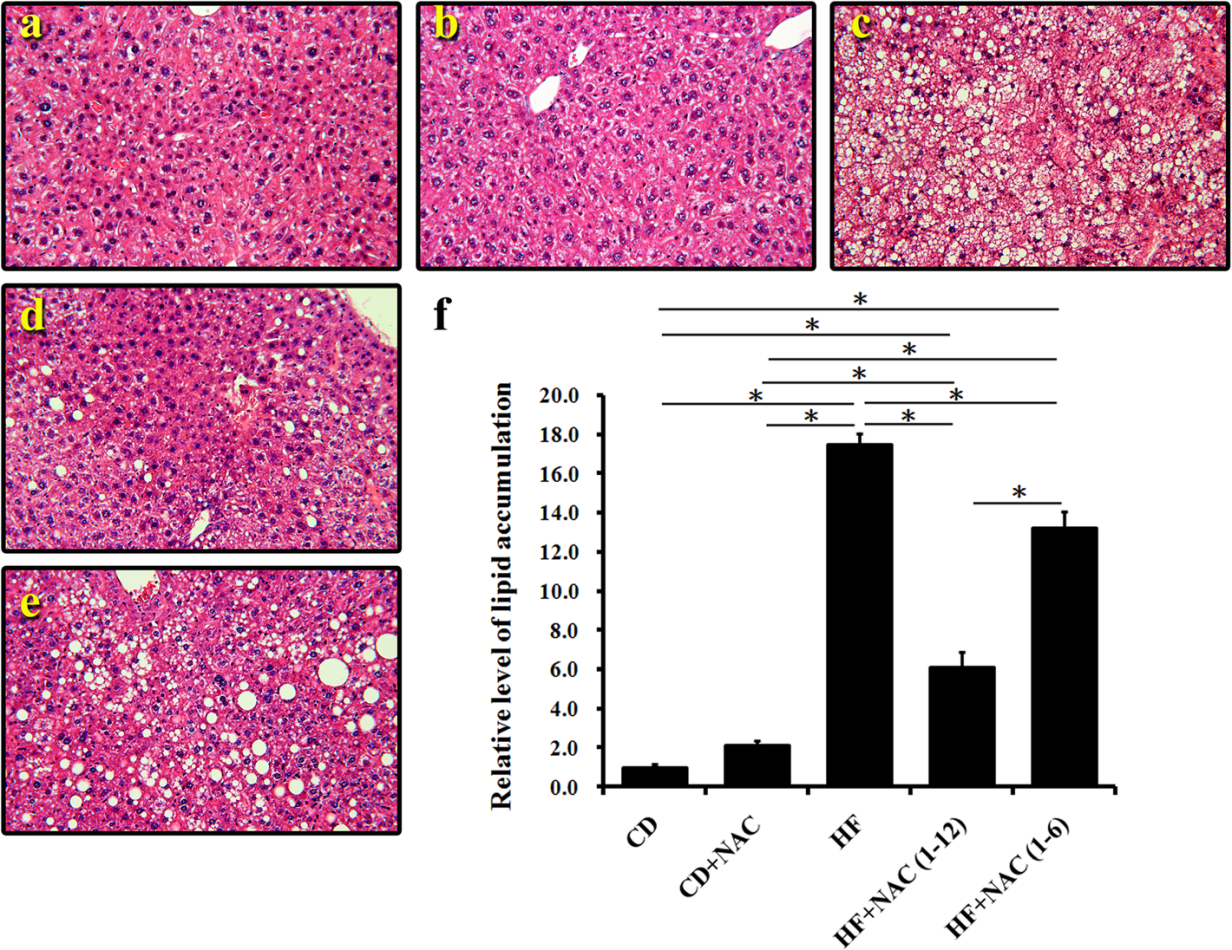
Liver steatosis. H&E staining demonstrated the highest lipid accumulation in the high fat diet group (HF). Lipid accumulation in the HF + NAC (1–12) group was lower than in the HF and HF + NAC (1–6) groups. **a** chow diet group; **b** chow diet and NAC intervention 1–12 month group; **c** high fat diet group; **d** high fat diet and NAC intervention 1–12 months group; **e** high fat diet and NAC intervention 1–6 months group; **f** quantification (original magnification × 200, *n* = 6)

Liver steatosis was assessed using H & E staining. Liver lipid accumulation increased in the HF group compared to the chow only, and chow with NAC diets (Fig. 2a, b, c). Lower lipid accumulation was observed in the HF + NAC(1–12) group than in the HF and HF + NAC(1–6) groups (Fig. 2d, e). NAC treatment for 12 months (full intervention) and 6 months effectively (fully) and partially improved liver fat accumulation caused by HF diet, respectively. The semi-quantitation is shown in Fig. 2f.

### Lipogenesis and fatty-acid oxidation

**Fig. 3:**
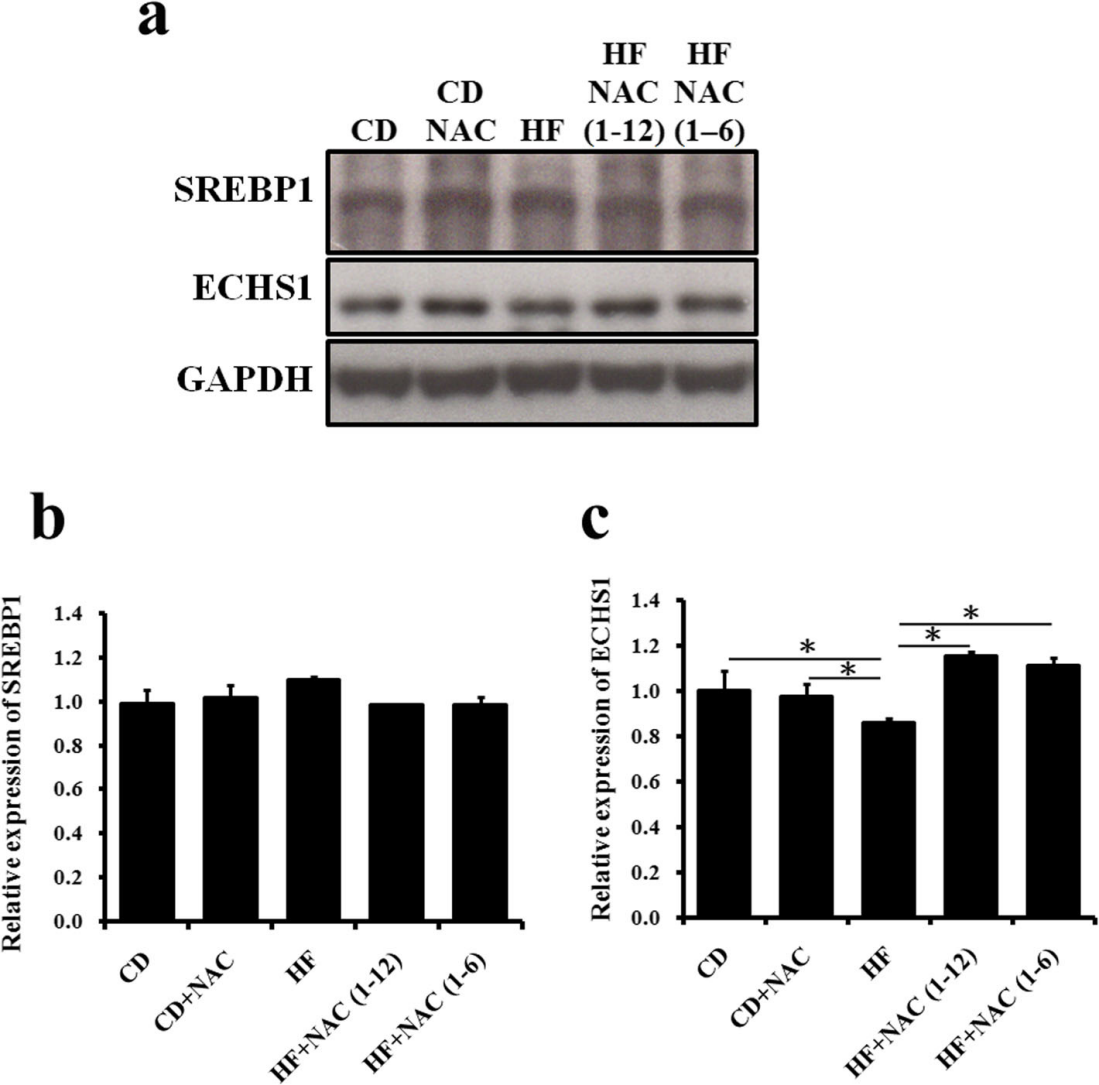
ECHS1 protein expression was highest in the HF + NAC (1–12) group; however, SREBP1 levels did not differ among the groups. **a** Western blot results for SREBP1 and ECHS1; quantification of **b** SREBP1 and **c** ECHS1 expression. The letters represent different groups (CD for chow diet group; CD + NAC for chow diet and NAC intervention 1–12 months group; HF for high fat diet group; HF + NAC (1–6) for high fat diet and NAC intervention 1–6 months group; HF + NAC (1–12) for high fat diet and NAC intervention 1–12 months group, all values are expressed as mean ± SEM (*n* = 6). *P < 0.05

Sterol regulatory element-binding protein 1 (SREBP1) is an important transcription factor regulating glycolysis and lipogenesis [20]. No differences in the expression of the SREBP1 active form were observed after HF diet or NAC (6 or 12 months) administration (Fig. 3a, b). Thus, lipogenesis may not be a major pathway for liver fat accumulation caused by a HF diet. The ECHS1 protein is the key enzyme for the second step of fatty-acid oxidation [21, 22]. We observed significantly reduced enoyl-CoA hydratase (ECHS1) levels in the HF group (Fig. 3a, c). Furthermore, both 12 and 6 months of NAC treatment effectively increased ECHS1 expression (Fig. 3a, c); the full course NAC administration effect was not more pronounced than that of 6 months NAC treatment (Fig. 3a, c). Overall, NAC was effective in reducing liver fat accumulation, possibly through restoring fatty-acid oxidation in hepatocytes and increasing fat metabolic capacity.

### Liver cell apoptosis via TUNEL staining and caspase 3 protein expression

**Fig. 4:**
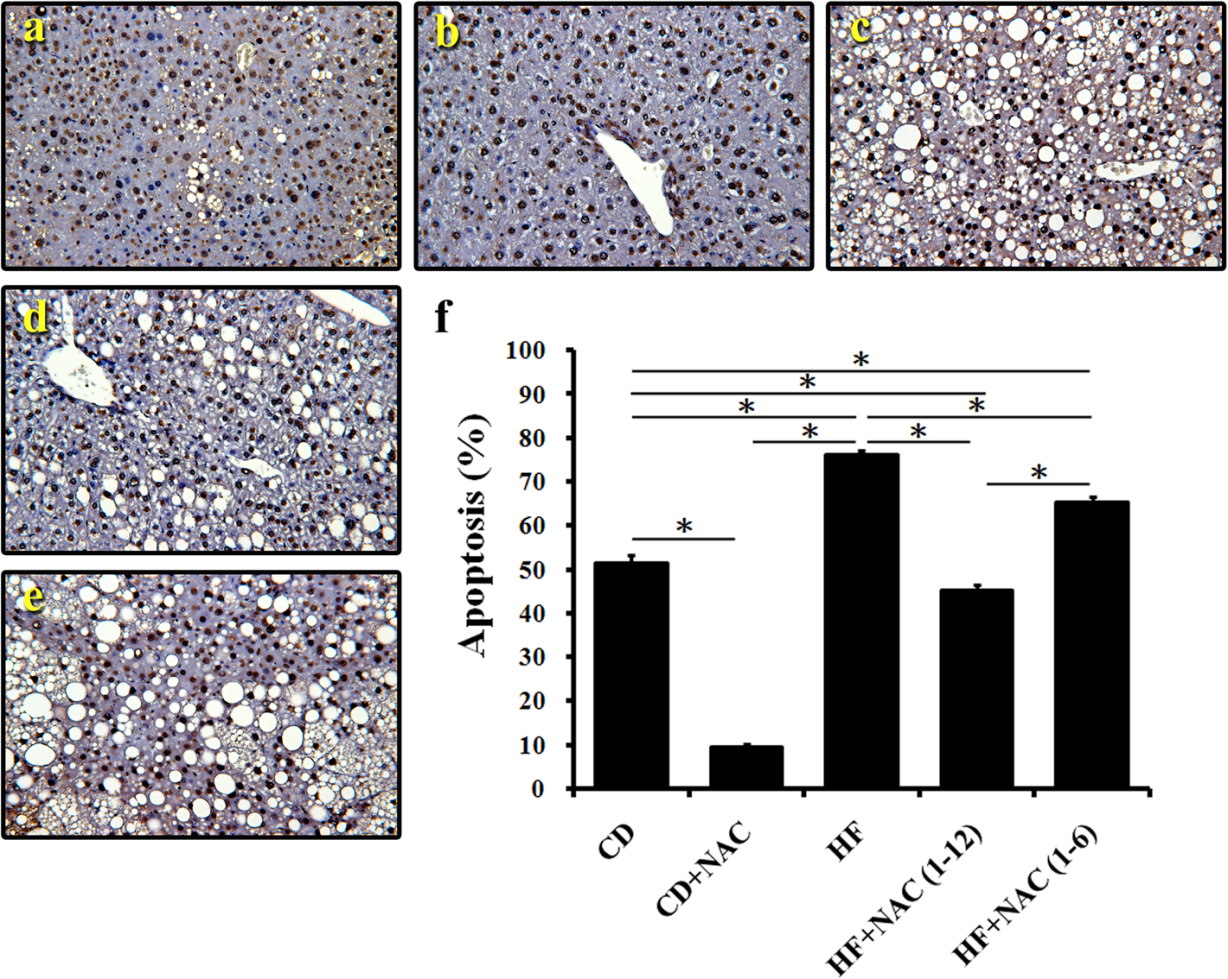
The extent of TdT-mediated dUTP biotin nick end labeling (TUNEL) staining was determined as an indicator for apoptosis. TUNEL staining showed the highest apoptosis levels in the high fat diet group (HF); the HF + NAC (1–12) group had lower apoptosis levels than the HF and HF + NAC (1–6) groups. **a** chow diet group; **b** chow diet and NAC intervention 1–12 month group; **c** high fat diet group; **d** high fat diet and NAC intervention 1–12 months group; **e** high fat diet and NAC intervention 1–6 months group; **f** quantification (original magnification× 200, *n* = 6)

**Fig. 5:**
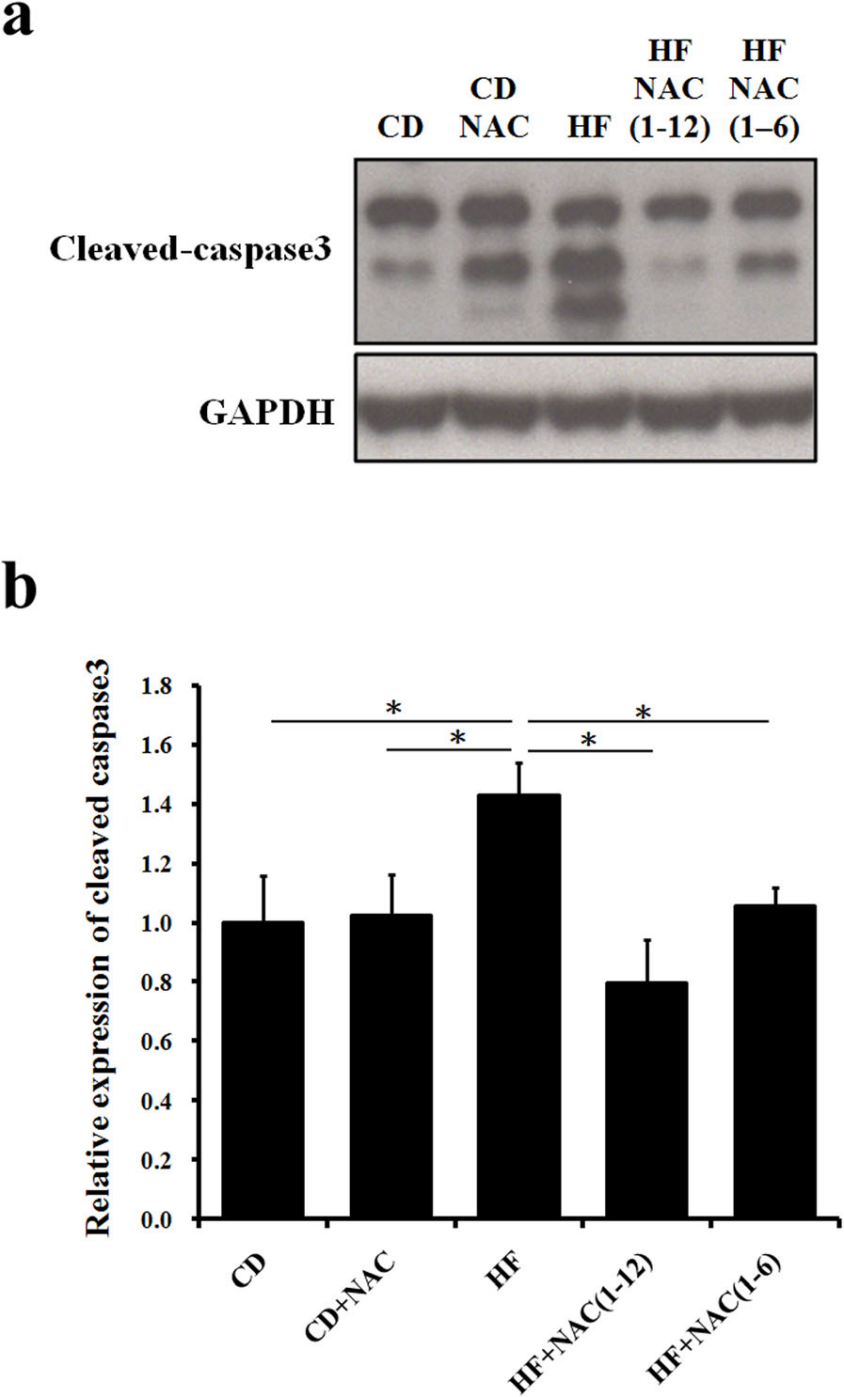
Apoptosis analysis. The highest cleaved caspase-3 protein expression, indicative of apoptosis, was detected in the high fat diet group (HF). The expression of cleaved caspase-3 in HF + NAC (1–12) group was lower than the HF group. **a** Western blot results of cleaved caspase3. **b** quantification of cleaved caspase-3 expression. The letters represented different groups (CD for chow diet group; CD + NAC for chow diet and NAC intervention 1–12 months group; HF for high fat diet group; HF + NAC (1–6) for high fat diet and NAC intervention 1–6 months group; HF + NAC (1–12) for high fat diet and NAC intervention 1–12 months group, all values are expressed as mean ± SEM (*n* = 6). *P < 0.05

According to our previous research results, excessive fat accumulation in the liver triggers liver cell apoptosis [14]. When cells undergo apoptosis, incomplete chromosomal DNA breaks occur, which can be detected by TUNEL staining. In addition to promoting liver fat accumulation, the HF diet also induced apoptosis compared to the chow only and chow with NAC diets (Fig. 4a, b, c, f). The full course NAC(1–12) treatment reduced liver cell apoptosis most effectively (Fig. 4d, f), whereas 6 months NAC(1–6) treatment still had a partial therapeutic effect (Fig. 4e, f). A similar trend was observed for the expression of cleaved caspase-3, an indicator of apoptosis. HF diet significantly increased cleaved caspase-3 expression, but the effect of 12 months NAC treatment on cleaved caspase-3 was the same as that of 6 month NAC administration (Fig. 5a, b).

### ER stress and UPR pathway

**Fig. 6:**
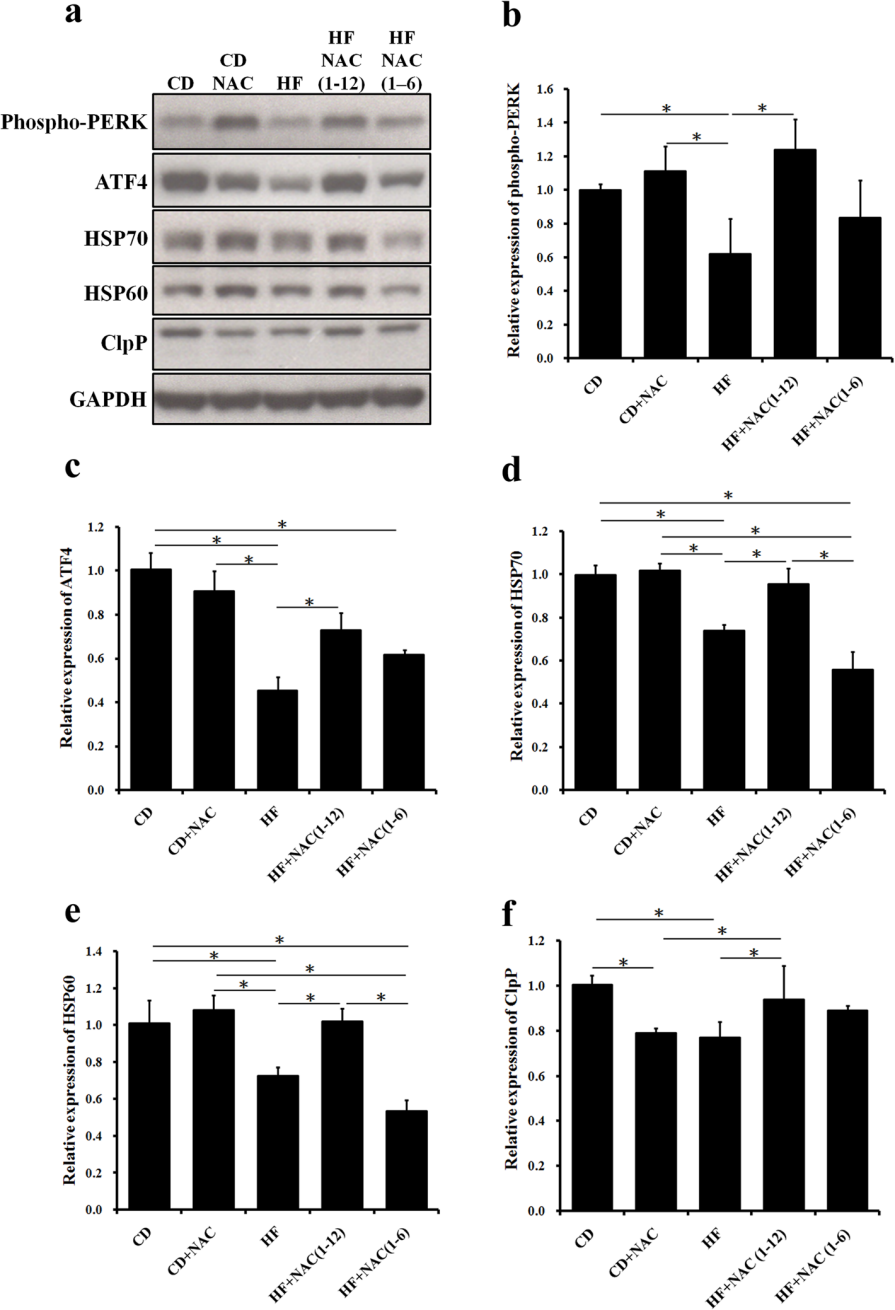
ER stress and UPR pathway are suppressed in the HF group. URP-related protein levels are significantly increased in the HF + NAC (1–12) group. **a** Western blot results for p-PERK, ATF4, HSP70, HSP60, and ClpP. Quantification of **b** p-PERK, **c** ATF4, **d** HSP70, **e** HSP60, and **f** ClpP expression. All values are expressed as mean ± SEM (*n* = 6). *P < 0.05. The letters represent different groups (CD for chow diet group; CD + NAC for chow diet and NAC intervention 1–12 months group; HF for high fat diet group; HF + NAC (1–6) for high fat diet and NAC intervention 1–6 months group; HF + NAC (1–12) for high fat diet and NAC intervention 1–12 months group)

**Fig. 7:**
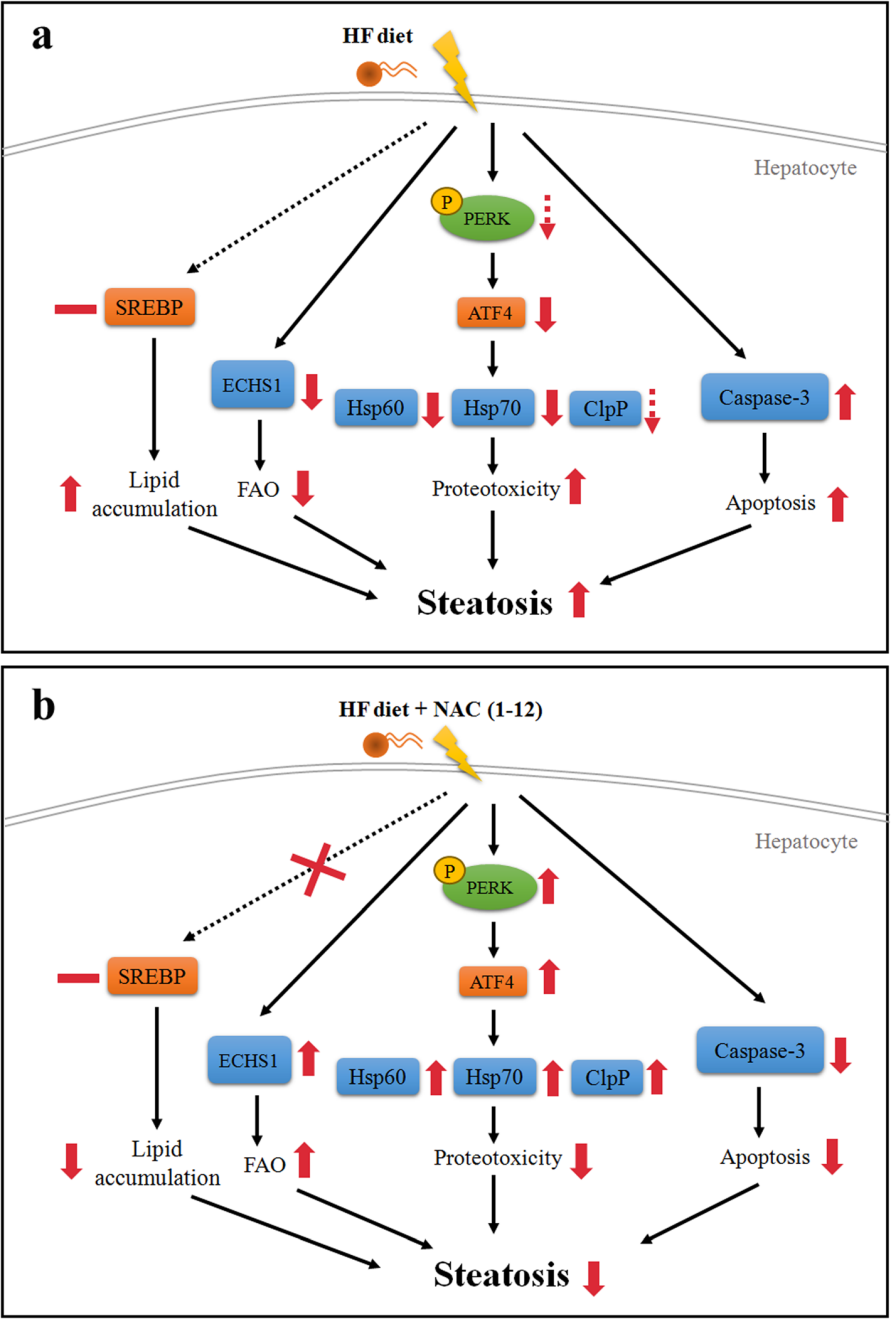
Scheme for studied pathways. **a** HF diet group. **b** HF + NAC(1–12) group. Red solid and dotted line stands for significant and non-significant difference, respectively

Oxidative and endoplasmic reticulum (ER) stress may be the main causes of liver cell apoptosis [14, 23]. ER is one of the main organelles for protein synthesis, folding, or modifications. Excessive protein misfolding may induce ER stress and unfolded protein response (UPR), which is activated by increasing the fat metabolic capacity by p-PERK on the endoplasmic reticulum membrane. It also promotes the downstream ATF4 to enter the nucleus and regulate the expression of UPR-reactive proteins, such as chaperones (heat shock protein (HSP)). The UPR reaction inhibits the transcription of new proteins; furthermore, it increases chaperone protein levels to facilitate the correct folding and protease clearance of misfolded proteins and to maintain the normal physiological function of the cells. Our results showed that a long-term HF diet significantly inhibits the expression of p-PERK and the downstream ATF4. (Fig. 6a, b, c) Furthermore, HSP70 and HSP60 functions were significantly increased by HF + NAC and HF + NAC(1–12) treatment when comparing to HF. (Fig. 6a, d, e) The increase p-PERK, HSP70 and HSP60 were increased in HF + NAC(1–12) with unfolded protein response not in HF + NAC(1–6) as in Fig. 6b, d and e. In addition, the expression of the enzyme ClpP was also inhibited in the HF group compared to the chow. (Fig. 6a, f) In the NAC(1–12) group, the expression of p-PERK, ATF4, HSP70, HSP60 and ClpP was significantly increased; however, the NAC(1–6) treatment had no effect compared to the HF group (Fig. 6a, b, c, d, e, f). There was no significant difference of ClpP proteins between NAC(1–12) and NAC(1–6) (Fig. 6a, f). The up-regulated and down-regulated pathway scheme as Fig. 7.

## Discussion

NAC exerts protective effect against NAFLD in rats, including prevention of cytokine-induced hepatocyte damage and abnormal liver enzyme and indices [24]. Furthermore, lifelong NAC administration normalizes oxidative stress, prevents acetaminophen-induced liver failure, and inhibits hepatocyte apoptosis [9]. NAC protects mouse liver injury [10]. In our study, we proved that 12 months NAC administration decreases lipid accumulation, apoptosis, and ER stress damage in hepatocytes as depicted in Fig. 7a, b. Shen et al. have identified the beneficial effect of early NAC administration in HF mice, comparing the effects of HF + NAC(1–6) and HF + NAC(3–6), including decreasing the weight of subcutaneous and visceral fat mass in adipose tissues. Impaired glucose tolerance test, increased oxidative stress, adiposity, and body fat were present to a higher degree in the late intervention in comparison with the early intervention [4]. Even though Shen et al. clarified the optimal timing of the NAC administration, they did not determine the necessary duration. In our study, we compared the effects of NAC(1–6) and NAC(1–12) and showed that longer NAC treatment duration is associated with more efficient reversal of liver lipid accumulation. Therefore, longer NAC treatment regimens should be considered in the future.

According to Oliveira, under the use of NAC for 4 weeks, there are several significant changes in metabolism and liver function, including 5.8% decrease in body weight, 15% decrease in serum cholesterol, 33% decrease in serum triglyceride, 90% decrease in ALT and 70% decrease in AST, respectively [25]. This study also demonstrated that NAC down-regulated the Fatty acid metabolism, oxidative phosphorylation and apoptosis. When it comes to compare the effect of NAC with other approaches causing restriction of weight gain, one small prospective study had compare the effects of NAC and Metformin on women with polycystic ovarian syndrome (PCOS). In 45 women with PCOS, there is significant improvement of body mass index, waist circumference, and weight reduction in patient using NAC, but no obvious different effect between NAC and Metformin [26]. There is still no large convincing prospective study to compare the effect of NAC to other possible approaches in weight reduction at present.

Regarding the causes of liver fat accumulation after a HF diet, there may be three pathways inducing fat accumulation in the liver. First, excess energy is converted into triglycerides for storage via lipogenesis. Second, fatty-acid oxidation (or β-oxidation), through which mitochondria are involved in the metabolism of fatty acids, is inhibited; consequently, liver cells do not consume excess fat effectively and cause lipid accumulation. Third, adipose tissue exports a large number of triglycerides to the liver via the blood circulation; however, the liver cannot process them causing fat accumulation. We detected SREBP1 and ECSH1, which are key proteins implicated in liver lipogenesis; furthermore, the ECSH1 protein participates in liver steatosis [21, 22]. Fatty acids are metabolized by fatty-acid oxidation into the final product acetyl-CoA in the TCA cycle and converted to ATP [27]. The results may be representative of a long-term HF diet inhibiting the liver ability to regulate excess fat into energy and further worsening liver fat accumulation.

Steatosis may be caused by the interaction of diet, genetic factors, gut microbiota, and lipogenesis through upregulation of lipogenic transcription factors, such as SREBP1c, etc. ER stress induces gluconeogenesis enzymes and activates SREBP, which is responsible for lipid accumulation in the liver [28]. SREBP1 is one of the transcription factors regulating hepatic lipid synthesis [29]. Moreover, SREBPs drive transcriptional programs increasing cellular cholesterol synthesis and import [30]. Both HF + NAC(1–12) and HF + NAC(1–6) did not change SREBP1 levels in our study. NAC may not regulate the lipid metabolism through SREBP1.

ECHS1, which has been first detected in ox heart and liver, catalyzes the conversion of trans-Δ2-enoyl-CoA thioesters to 3-L-hydroxyacyl-CoA thioesters and is responsible for the second step of fatty acid β-oxidation (FAO) [21, 22]. ECHS1 has moderate activity for methacrylyl-CoA (valine pathway), 3-methylcronytyl-CoA (leucine pathway), and tiglyl-CoA (isoleucine pathway) degradation [31, 32]. Elevated ECHS1 protein expression was noted in both HF + NAC(1–6) and HF + NAC(1–12) in comparison with the HF diet, indicating that FAO may be increased with NAC use longer than 6 months to prevent liver steatosis progression.

DNA damage quantification by TUNEL is widely utilized for cellular apoptosis and drug toxicity assessment [33]. Cysteine protease (caspase) activation is the most useful biochemical hallmark of both early and late apoptosis stages. Therefore, active caspase-3 detection in cells and tissues is an important method for apoptosis determination [34]. We found significantly lower apoptosis rate in the HF + NAC(1–12) group than in the HF + NAC(1–6) using TUNEL, suggesting longer NAC use has better anti-apoptotic effect in liver lipid accumulation. However, there is no difference between these two NAC groups in caspase-3 assays.

ClpP, a mammalian quality control protease, plays an important role in the initiation of the mitochondrial UPR, maintaining mitochondrial protein homeostasis [35]. However, several studies revealed that ClpP loss may trigger compensatory responses in mice and provide metabolic benefit [35, 36]. One investigation suggested that ClpP may be dispensable for mammalian UPR initiation [35]. In our study, ClpP was elevated in the HF + NAC(1–12) but not HF + NAC(1–6) group, which may be associated with sequential decrease of mitochondrial dysfunction and metabolic disorders.

ATF4 and p-PERK sequentially activate autophagy, which can prevent NAFLD progression in mice via an ATF4-dependent pathway [37]. According to Kim et al., carbon monoxide induces the p-PERK-eIF2a-ATF pathway and is a potentially effective strategy to prevent the progression of NAFLD [37]. In our study, ATF4 was significantly elevated in NAC(1–12), which could alleviate the progression of NAFLD through decreasing lipotoxicity.

HSP70, which is induced by stress including ischemia, infection, inflammation, and exposure to organics and oxidants, improves cell survival by protecting cells from proteotoxicity [38]. Therefore, our finding that HSP70 is decreased and elevated in the HF + NAC(1–6) and HF + NAC(1–12) groups, respectively, can indicate that NAC should be used for 12 months to increase HSP70 levels and promote cell survival. In addition, HSP60 has been associated with oxidative stress and its down-regulation by NAC has been demonstrated [39]; however, in our study HSP60 was up-regulated by NAC in the HF + NAC(1–12) but not the HF + NAC(1–6) group. Prolonged NAC use (12 months) may be beneficial for decreasing liver lipid accumulation and apoptosis, possibly via HSP60 and HSP70 upregulation reducing proteotoxic stress.

## Conclusion

Long-term HF diet of newborn offspring causes liver steatosis and inhibits the protective effect of UPR induced by ER stress with hepatocyte apoptosis. NAC administration reduces liver fat accumulation, effectively restores the protective effect of UPR, and reduces hepatocyte damage and apoptosis. The protective effect of NAC is more pronounced with a long term administration.

## Data Availability

The datasets used and analyzed during the current study are available from the corresponding author upon a reasonable request.

## Abbreviations

NAFLD: Non-alcoholic fatty liver disease
ER: Endoplasmic reticulum
ROS: Reactive oxygen species
NAC: N-acetylcysteine
GSH: glutathione
HF: high fat diet
CD: chow diet
PVDF: polyvinylidene difluoride
ECHS1: Enoyl-CoA hydratase
SREBP1: Sterol regulatory element-binding protein 1
UPR: unfolding protein response
p-PERK: protein kinase RNA-like endoplasmic reticulum kinase phosphorylation
ATF4: activating transcription factor 4
HSP: heat shock protein
ClpP: Clp protease proteolytic subunit
HIRI: hepatic ischemia-reperfusion injury
JNK: c-Jun N-terminal kinase
FAO: fatty acid β-oxidation
